# Chemical composition and antifungal activity of *Capsicum* pepper aqueous extracts against plant pathogens and food spoilage fungi

**DOI:** 10.3389/fcimb.2024.1451287

**Published:** 2024-10-03

**Authors:** Marcela Sepúlveda, Jéssica Costa, Yasna Cayún, Víctor Gallardo, Elsa Barría, Glaucia Rigotto Caruso, Marcia Regina von Zeska Kress, Pablo Cornejo, Cledir Santos

**Affiliations:** ^1^ Doctorado en Ciencias de Recursos Naturales, Universidad de La Frontera, Temuco, Chile; ^2^ Departamento de Biologia, Instituto de Ciências Biológicas-ICB, Universidade Federal do Amazonas, Manaus, Amazonas, Brazil; ^3^ Department of Chemical Science and Natural Resources, Universidad de La Frontera, Temuco, Chile; ^4^ Programa de Pós-Graduação em Biotecnologia, Universidade Tecnológica Federal do Paraná, Ponta Grossa, Paraná, Brazil; ^5^ Department of Clinical Analysis, Toxicology and Food Sciences, School of Pharmaceutical Sciences of Ribeirão Preto, University of São Paulo, Ribeirão Preto, São Paulo, Brazil; ^6^ Escuela de Agronomía, Facultad de Ciencias Agronómicas y de los Alimentos, Pontificia Universidad Católica de Valparaíso, Quillota, Chile; ^7^ Centro Regional de Investigación e Innovación para la Sostenibilidad de la Agricultura y los Territorios Rurales (CERES), Quillota, Chile

**Keywords:** biofungicides, capsaicinoids, chilli pepper, phenolic compounds, secondary metabolite

## Abstract

*Capsicum* pepper is a rich source of phytochemical compounds such as capsaicinoids, phenols, flavonoids, and so forth. Due to their antimicrobial and antioxidant potential all of these compounds have been assessed and used for both human and plant health benefits. Herein, three fresh varieties of *Capsicum annuum* (Cacho de Cabra, Bell pepper, and Hungarian Wax varieties) and one fresh and ripe variety of *C. baccatum* (Cristal) were evaluated. Capsaicin, dihydrocapsaicin, nordihydrocapsaicin and the phenolic content of *Capsicum* spp. extracts were characterised. The antifungal potential of capsaicinoids and antioxidant activities, and the ecotoxicity of each *Capsicum* spp. extract, using the model *Galleria mellonella*, were also evaluated. Phytochemical analyses showed that the Cristal and Hungarian Wax varieties presented the highest amount of capsaicin, dihydrocapsaicin, and nordihydrocapsaicin; while Bell Pepper had the highest phenol content and antioxidant activity. Capsaicinoids’ standards and *Capsicum* spp. extracts showed fungistatic activity against the fungal strains assessed. For the fungal strains assessed, the fungistatic activities of capsaicinoids’ standards were higher than those observed in *Capsicum* spp. extracts. The Hungarian Wax extracts inhibited slightly the growth of *Aspergillus niger* MUM05.11 and *Fusarium oxysporum* MUM16.143. Similarly, *A. niger*, *F. oxysporum*, *Rhizopus arrhizus* MUM16.05 and *Alternaria* sp. UFRO17.178 had their growth retarded by the use of Cacho de Cabra and Cristal extracts. Noticeable changes were observed in the fungal strains’ morphologies, such as the presence of fragile fungal structures, pigmentation loss, variation in the reproductive structures size and the conidia number. *Capsicum* extracts weaken the growth of fungi, indicating their fungistatic potential. Considering the fungistatic potential and non-ecotoxicity of these extracts, it is possible to suggest their use as a tool for pest management in the agri-food sector, controlling the growth and reproduction of fungi without posing a risk to non-target biodiversity.

## 1 Introduction

The *Capsicum* L. genus encompasses herbaceous plant which belongs to the Solanaceae family (Govindarajan and Salzer, 1985; Motti, 2021). This plant genus comprises more than 30 species, from which *Capsicum annuum*, *C. baccatum*, *C. chinense*, *C. frutescens* and *C. pubescens* are the most common ones. *Capsicum* pepper is original from intertropical America. Currently, it is a horticultural crop widely cultivated throughout the world (Govindarajan and Salzer, 1985; Costa et al., 2019b, 2020, b).

In Chile, *C. annuum* and *C. baccatum* are the most produced and consumed species (Costa et al., 2020, b). These species cover landraces of peppers (e.g., Cristal, Largo Cayenne and Anaheim) with various shapes, sizes and fruit colours (e.g., yellow, orange, green and red), including spicy and non-spicy varieties; the last one also known as sweet peppers (Costa et al., 2020; Tobolka et al., 2021; Costa et al., 2022a, b).

*Capsicum* pepper can be eaten fresh, dried, crushed, powdered and mixed with other spices, as a vegetable in the case of the sweet pepper varieties and as a spicy condiment in the case of the pungent varieties. The sweet peppers varieties (e.g., Hungarian sweet wax) are used for the preparation of pickles and paprika. While pungent varieties (e.g., Crystal, Cacho de Cabra, Cayenne S Largo, Anaheim and Chilean) are the most cultivated and consumed due to their aroma and spiciness (FIA, 2006).

*Capsicum* peppers are a rich source of bioactive metabolites such as carotenoids, fatty acids (e.g., α-linolenic acid, linoleic acid, and palmitic acid), phenolic compounds (e.g., flavonoids, phenolic acids), and capsaicinoids (Fratianni et al., 2020; Leng et al., 2022). The differences in plant genotypes, fruit ripeness, harvesting conditions and the environment can affect the content and profiles of these bioactive metabolites in *Capsicum* plants (Saini et al., 2021; Costa et al., 2022b).

Carotenoids are responsible for the colours of pepper as well as phenolic compounds. The capsaicinoids are responsible for fruit pungency and play a role in plant defence mechanisms against predators and pathogens (Tewksbury and Nabhan, 2001; Adams et al., 2020; Chabaane et al., 2022).

The capsaicinoids are secondary metabolites classified as phenolic alkaloids, which are present in different amounts in *Capsicum* fruits (Naves et al., 2019). Within these alkaloids, capsaicin (CAP) and dihydrocapsaicin (DHC) can predominantly be found, representing 77 to 98% of capsaicinoids, followed by nordihydrocapsaicin (n-DHC), and homo-dihydrocapsaicin (h-DHC) which are found in low amounts (Stephen and Kumar, 2014; Tobolka et al., 2021; Waqas et al., 2022; Costa et al., 2022b).

The capsaicinoids from *Capsicum* pepper extracts are natural products that can be applied to control the growth in plant crop production of both phytopathogen and spoilage fungi. Previous reports point out that *Capsicum* extracts were effective against phytopathogen and spoilage fungi such as *Alternaria alternata*, *Botrytis cinerea*, *Cladosporium* sp., *Fusarium oxysporum*, *Fusarium* sp., *Penicillium* sp., *Phytophtora capsica*, *Rhizoctonia* sp., *Verticillium dahlia* (Kraikruan et al., 2008; Singh and Chittenden, 2008; Singh et al., 2011; Fieira et al., 2013; Zanotto et al., 2016; Kollia et al., 2019; Buitimea-Cantúa et al., 2020; Saini et al., 2021; Vázquez-Fuentes et al., 2021; Hernández-Téllez et al., 2022; Nidiry, 2022; Valencia-Hernandez et al., 2022).

The capsaicinoids’ potential as a biofungicides could open up new avenues for an environmentally eco-friendly horticulture management (Costa et al., 2022b). Biofungicides can be applied from seed to cultivars, promoting protection against fungal pathogens (Costa et al., 2022b). In addition, the metabolites of *Capsicum* peppers (e.g., phenolic compounds, capsaicinoids) show antioxidant potential (Costa et al., 2022b). The exogenous application of compounds/extracts with antioxidant activity can improve plant physiology and development, being especially important in conditions of abiotic stress (e.g., salt stress, drought, high temperatures) (Shalata and Neumann, 2001; Shi et al., 2010, 2015; Singh et al., 2015; Queiroz et al., 2023). This field of application has not yet been explored with capsaicinoids and *Capsicum* metabolites.

In Chile, despite the high consumption of chilli peppers, there are a little information on the profile of these molecules in *Capsicum* species and their landraces (Lutz et al., 2015; Riquelme and Matiacevich, 2017; Muñoz-Concha et al., 2020). The establishment of phenolic and capsaicinoid profiles is essential, as these metabolites can be used to develop bioproducts with agricultural applications. In addition, these molecules add commercial value to peppers, contributing to the aroma, colour and flavour, showing a great benefit to the end consumer’s health (Baenas et al., 2018).

The main aim of this work was to quantify the capsaicin, dihydrocapsaicin, nordihydrocapsaicin and the phenolic content of aqueous extracts of four *Capsicum* spp. varieties. In addition, the antifungal potential of capsaicinoids against phytopathogen and spoilage filamentous fungi and the ecotoxicity of each *Capsicum* spp. extract, using the model *Galleria mellonella*, were also assessed.

## 2 Materials and methods

### 2.1 Plant material

**Figure 1:**
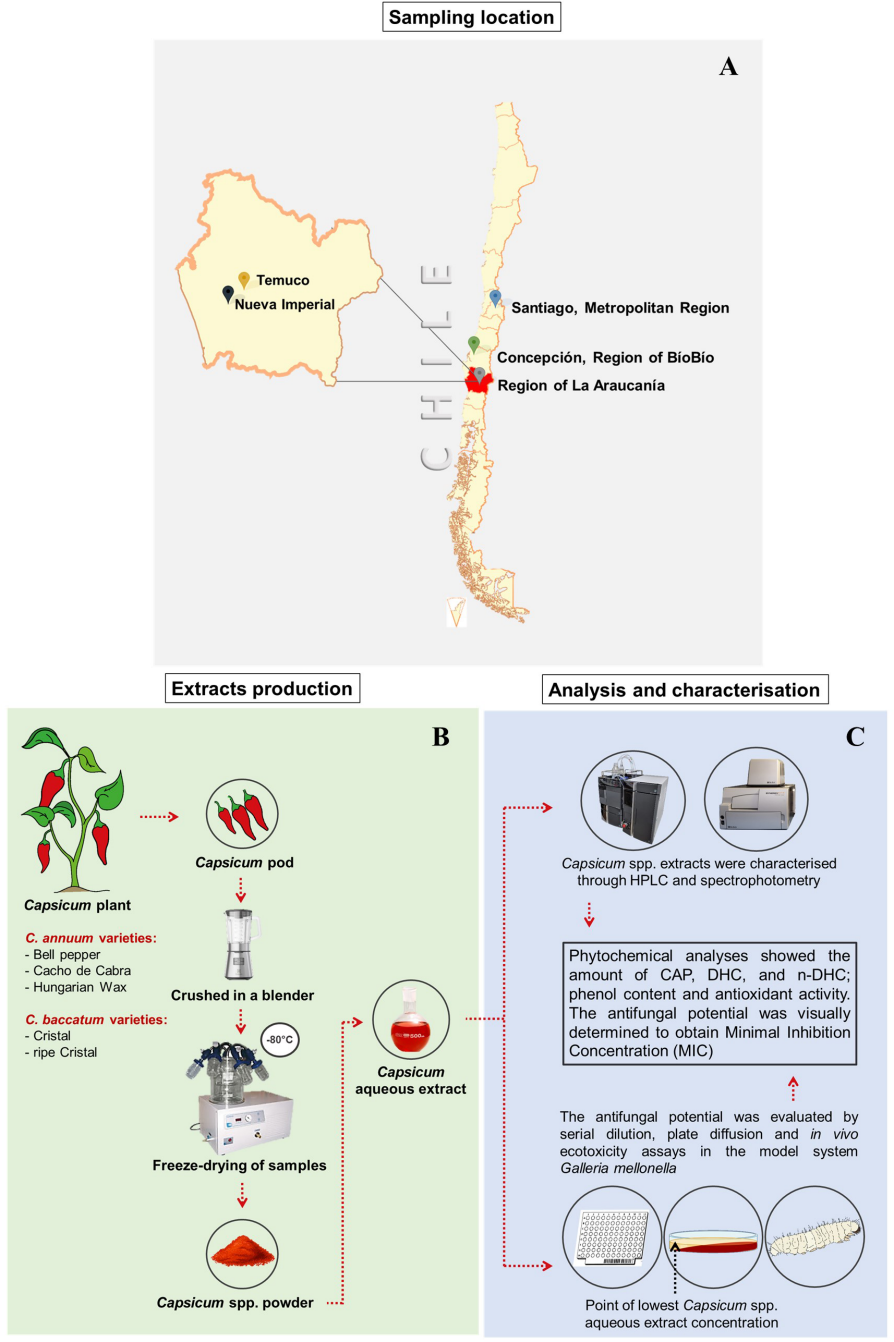
Schematic representation with the map of the geographic place of pepper sampling **(A)**, the procedure used in *Capsicum* spp. extracts’ production **(B)**, analyses and antifungal and ecotoxicity assessments **(C)**.

For this study, three fresh varieties of *Capsicum annuum* (Cacho de Cabra, Bell pepper, Hungarian Wax) and a green and a ripe sample of *C. baccatum* (Cristal variety) were evaluated. Samples were purchased from both a local market in Temuco city, capital of the Region of La Araucanía, Chile and from a farmer based on the countryside of Nueva Imperial city (Region of La Araucanía, Chile) (**Figure 1A**).

Pepper samples were separately immersed in 0.3% NaClO aqueous solution for 1 minute and rinsed with distilled water. After removing the excess water with a gauze, the chilli peppers were cut into large pieces discarding the pedicel. Each chilli pepper sample was crushed in a blender until it formed a puree. The chilli purees were frozen at -80°C and then freeze-dried for 5 days in a L101 Liobras freeze-dryer (São Carlos, SP, Brazil). Subsequently, all samples were stored in Schott vials at -80°C for later analysis (**Figure 1B**).

### 2.2 Microorganisms

The fungal cultures used in this study were *Alternaria* sp. UFRO17.178, *Aspergillus niger* MUM05.11, *Aspergillus dimorphicus* UFRO17.168, *Fusarium oxysporum* ATCC48112, *Fusarium verticillioides* MUM16.143, *Penicillium citrinum* UFRO17.97 and *Rhizopus arrhizus* MUM16.05. All fungal strains were obtained from the Bank of Microbiological Resources of the Universidad de La Frontera (Temuco, Chile) and the Micoteca da Universidade do Minho (Braga, Portugal).

### 2.3 Preparation of aqueous extracts of *Capsicum* spp.

The extraction procedure was based on the methodology previously described elsewhere with modifications (Koffi-Nevry et al., 2012). Briefly, a sample composed of 20 g of each freeze-dried *Capsicum* spp. puree was mixed with 300 mL of distilled water. The mixture was heated in a water bath at 90°C for 20 minutes with periodic agitation. The extract was filtered in a double layer of gauze and dried by a rotary evaporator. The crude obtained was stored at 4°C for further analysis (**Figure 1B**).

### 2.4 Capsaicinoids quantification by HPLC-FD

The determination of capsaicin, dihydrocapsaicin, and nordihydrocapsaicin on aqueous extracts of *Capsicum* spp. were performed on a High-Performance Liquid Chromatography with Fluorescence Detection system (HPLC-FD, Shimadzu, Japan) equipped with a InertSustain C18 (4.6 × 250 mm, 5 μm) column and a GL-Cart Cartridge pre-column, a fluorescence detector (RF-20A, Shimadzu Co. Kyoto, Japan), LC-40D pump, RF-20A fluorescence detector (FD), CTO- 40C column oven and DGU-405 solvent degasser. For capsaicinoid peak detection operating conditions included a flow rate of 1 mL/min, and a run duration of 20 min. The mobile phase was a gradient consisting of 57% solvent B (100% methanol) and 43% solvent A (10% methanol in water) for 10 min followed by 68% solvent B and 32% solvent.

A for an additional 10 min. The eluting compounds were monitored by fluorescence detection with excitation at 265 nm and an emission cut-off of 320 nm.

The standards of CAP, DHC and n-DHC (≥ 95% of purity from *Capsicum* sp.) were obtained from Sigma-Aldrich (St. Louis, MO, USA). All solutions prepared for HPLC were passed through 0.45 µm nylon membrane-size filters previous to use.

### 2.5 Extraction of phenolic compounds

The extraction was carried out with the method previously described elsewhere with modifications (Nahuelcura et al., 2022). An aliquot of 0.3 g of freeze-dried *Capsicum* spp. puree was crushed and mixed with 3 mL of the extraction solvent (methanol:formic acid) (95:5, v/v) in the dark. The samples were sonicated with an ultrasonic bath for 60 s, shaken for 30 min at 200 x g and centrifuged for 10 min at 4000 x g. The supernatants were stored at -20°C until use. The extracts’ solvents were then removed by using a rotary evaporator (Büchi, Flawil, Switzerland). Crude extracts were suspended in solution composed by water:acetonitrile:formic acid (92:3:5, v/v/v) and filtered through 0.45 µm filters. For this case, the volume as that recovered in the supernatant indicated above was used.

#### 2.5.1 Determination of phenolic compounds in *Capsicum* spp. extracts

The determination of total hydroxycinnamic acids (THCA), flavanols (TF) and phenolic acids (TPA) compounds was carried out according to Alarcón et al. (2022), with modifications. An HPLC (Shimadzu, Tokyo, Japan) equipped with an LC-20AT quaternary pump and a DGU-20A5R degassing unit, a CTO-20A oven and an SPD-M20A diode-array detector (DAD) was used. A C18 column (Kromasil 250 × 4.6 mm, 5 μm) equipped with a C18 guard column (22 × 3.9 mm, 4 μm; Novapak, Waters, Milford, MA, USA) was used. The oven temperature was 40°C. Gradient elution was performed with a flow rate of 0.8 mL/min using mobile-phase (A) water:acetonitrile:formic acid (87:3:10, v/v/v) (10:90) and (B) water:acetonitrile:formic acid (40:50:10, v/v/v). The chromatographic analysis was performed using a gradient as follows: mobile phase B from 6% to 20% at min 10, from 20% to 30% at min 15, from 30% to 50% at min 30, from 50% to 60% at min 35, from 60% to 6% at min 41 and, finally, from 6% to 0% at min 50. Quantification was performed using gallic acid, chlorogenic acid and quercetin standards at 280, 320 and 360 nm, respectively (**Figure 1C**).

#### 2.5.2 Total phenol assay

The total phenolic content (TPC) was determined using the Folin-Ciocalteu reagent. The analysis was performed in a microplate reader (Epoch™) using the methods optimised for 96-well plates described by Parada et al. (2019) with modifications. Briefly, 15 µL of standard or extract, 750 µL of deionised water, 75 µL of Folin–Ciocalteu reagent, 300 µL of sodium carbonate 20% w/v and 360 µL deionised water were added in an microtube. The solutions were incubated for 30 min, at 20°C in the dark. Then, 250 µL of the solution was added to the microplate wells. The absorbance was measured using a multi-mode microplate reader (Biotek Synergy™ HTX) at 750 nm. A gallic acid solution was used as a standard (**Figure 1C**).

### 2.6 Determination of antioxidant activity of *Capsicum* spp. extracts

For antioxidant activity, all assays were adapted to a microplate reader (Epoch™) and adjusted to small volumes as described by Parada et al. (2019). For all three methods, Trolox (6-hydroxy2,5,7,8-tetramethyl-chroman-2-carboxylic acid), obtained from Sigma-Aldrich (Steinheim, Germany), was used as the standard and the results were expressed as µmol Trolox/g.

#### 2.6.1 ABTS•+ (2.2’-azinobis 3-ethylbenzothiazoline 6-sulfonate) scavenging method

For the ABTS antioxidant activity method, a 7.5 mmol L^–1^ ABTS•+ stock solution was prepared as described by Kunnaja et al. (2021) with modifications. In 10 mL of HPLC-grade water, 38.4 mg of ABTS and 6.9 mg of potassium persulfate were mixed and dissolved. For radical activation, the stock solution was incubated for 24 h at 4°C in the dark to allow the reaction. After incubation, 685 μL of the ABTS•+ stock solution was diluted in 50 mL of 95% ethanol. For use, 245 μL of diluted ABTS+ 7.5 mmol L^–1^ and 5 μL of the standard or the sample was added to a 96-well plate. The solution was incubated for 30 min at 30°C. After this, the absorbance was measured using a multi-mode microplate reader at a wavelength of 734 nm.

#### 2.6.2 Copper-reducing antioxidant capacity

A mix containing 50 μL of 10 mmol L^–1^ CuCl_2_, 50 μL of 7.5 mmol L^–1^ neocuproine, and 50 μL of 1 mol L^−1^ ammonium acetate buffer pH 7 were added to a 96-well plate. This mixture was left to incubate for 15 min at 27°C, then the standard or sample was added and incubated for another 30 min at 27°C. The absorbance was measured at 450 nm.

#### 2.6.3 DPPH (2,2-diphenyl-1-pycrilhydrazil hydrate) scavenging method

A total of 240 μL of 0.1 mmol L^–1^ DPPH radical dissolved in methanol was added to a 96-well plate, 10 μL of sample or standard was incubated for 30 min and the measurements were taken at 517 nm using a multi-mode microplate reader.

### 2.7 Antifungal activity

#### 2.7.1 Inoculum preparation

The fungal strains *Alternaria* sp. UFRO17.178, *Fusarium oxysporum* ATCC48112, *F. verticillioides* MUM16.143 and *Rhizopus arrhizus* MUM16.05 were inoculated on Potato Dextrose Agar (PDA, 200 g L^−1^ of infusion from potatoes, glucose 20 g L^−1^, agar 15 g L^−1^) at 28°C for 5 days at the dark. *Aspergillus niger* MUM05.11, *A. dimorphicus* UFRO17.168 and *Penicillium citrinum* UFRO17.97 were grown on Malt Extract Agar (MEA, malt extract 20 g L^−1^, mycological peptone 1 g L^−1^, agar 20 g L^−1^, glucose 20 g L^−1^) at 25°C for 7 days at the dark. Suspensions of fungal inoculum were prepared in sterile distilled water and were filtered through sterile gauze. The cell counts were determined with a hemocytometer to 10^5^ conidia/mL.

#### 2.7.2 Minimal fungistatic concentration

The minimum fungistatic concentration was defined as the lowest concentration of an antifungal agent capable of modifying the normal fungal growth changing their macro- and micro-morphological characteristics, when compared with control (fungus + culture medium). The MFCs of each chilli pepper extract were determined by using both liquid dilution MFC assays and well diffusion assays. The broth microdilution tests followed the procedure for Minimum Inhibitory Concentration (MIC) established at the Clinical Laboratory Standards Institute (CLSI) guidelines (CLSI, 1995).

A stock solution of the chilli aqueous extracts was prepared in sterile distilled water up to 100 times the final concentration required. Further, the chilli aqueous extracts were diluted in Roswell Park Memorial Institute (RPMI) 1640 medium, pH 7.0 (Sigma-Aldrich Co., St. Louis Mo., USA) to obtain the ×2 concentration.

Each chilli pepper extract (100 mL) was transferred into a 96-well microdilution plate on separate wells on the top row of the plate. Samples of chilli pepper extracts or capsaicinoid standards (CAP, DHC and n-DHC) were serially diluted in each column of the plate, doubling the dilution. The conidial inoculum suspension (100 mL, containing approximately 10^5^ conidia/mL) was then added to all wells except the sterile control wells.

The final concentration of the chilli pepper solution was 0.39, 0.78, 1.56, 3.1, 6.25, 12.5, 25, 50, 100 and 200 mg/mL. For the standards, a final concentration of 0.48, 0.97, 1.9, 3.9, 7.81, 15.62, 31.25, 62.5, 125 and 250 µg/mL was obtained. A negative control (inoculum suspension and RPMI medium) and sterile control (broth without fungi) were also included on each plate.

For MFCs determination, microdilution plates were incubated at 28°C and visually examined from 0 up to 48 h time of inoculation.

#### 2.7.3 Well diffusion method

The test was carried out in Petri dishes with Sabouraud Dextrose Agar (SDA, 40 g/L glucose, 20 g/L peptone and 15 g/L agar). The *Capsicum* aqueous extracts were prepared according to the MFC value determined for each aqueous extract. The extracts were mixed with the SDA medium. Then, 4 mL of this solution was added to Petri dishes, which were left to solidify at a 45° angle. The plate was levelled and the SDA medium was added, forming a concentration gradient (**Figure 1C**).

One microlitre of fungal inoculum solution was placed at the point containing the lowest concentration of the *Capsicum* spp. aqueous extract as shown in the **Figure 1C**. The inoculated SDA medium was used as a positive growth control. The plates were incubated at 28°C for five days. Fungal inhibition was determined on each plate by comparing the growth diameters with the control. Macro and micro-morphological changes were also assessed.

### 2.8 Toxicity of pepper extracts

The toxicity of pepper varieties was evaluated using *Galleria mellonella* larvae, an invertebrate model of toxicity and virulence. The experiment was performed as described by Grizante-Barião et al. (2022). Briefly, the *Capsicum* extracts were suspended in distilled water. For each treatment, a group of 5 larvae of *G. mellonella* (200 to 250 mg) in the sixth instar of development were placed in sterile 6-cm Petri dishes. The doses tested for each *Capsicum* extract were 2000 mg/kg, 200 mg/kg and 20 mg/kg. As mortality control ethanol 99.9% was used; while distilled water and naïve larvae were used as survival controls (**Figure 1C**).

A Hamilton model 7000.5 KH micro-syringe was used to inject 5 μL of each extract into the hemocoel of each larva through the last right proleg. After inoculation, the larvae were kept at 37°C and deprived of food. The assessment of life and death was carried out every 24 hours, for 5 days. To delay metamorphosis pre-pupae were daily removed. Kaplan-Meier Survival curves were plotted using data pooled from a minimum of two independent experiments, the graphs were generated using the GraphPad Prism software, version 9.3.0 for Windows (San Diego, California, USA).

### 2.9 Data analysis

The data obtained were processed using R studio software (R version 4.3.1). The Shapiro-Wilk and Bartlett test was used to determine the normality and homoscedasticity of the data. Parametric (ANOVA test and Tukey’s *post-hoc* test) and non-parametric (Kruskal – Wallis test and Dunn’s *post-hoc* test) analyses were applied using an α value of 0.05. The analysis was performed using the stats package version 4.3.1 and the FSA package version 0.9.5.

## 3 Results and discussion

### 3.1 Capsaicinoids content

*Capsicum* pepper produces an array of capsaicinoids that are related to the fruit’s pungency. In this study, CAP, DHC, and n-DHC were identified and quantified by HPLC-FD analysis, and the results are shown in **Table 1**. For the calibration curve, standard solutions of 2 to 10 µg/mL of CAP, DHC and n-DHC were prepared in methanol. The retention times were 14.6 min for n-DHC, 15.1 min for CAP, and 17.8 min for DHC.

**Table 1:** Capsaicinoids content of different *Capsicum annuum* and *C. baccatum* varieties.

Variety	CAP (mg/g)	DHC (mg/g)*	n-DHC (mg/g)
Cacho de Cabra	1.35 ± 0.038c	1.19 ± 0.005ab	0.43 ± 0.003a
Hungarian Wax	2.24 ± 0.068b	1.41 ± 0.010ab	0.18 ± 0.023c
Bell Pepper	0.01 ± 0.001e	0.01 ± 0.003b	ND
Cristal	3.75 ± 0.051a	2.70 ± 0.017a	0.34 ± 0.006b
ripe Cristal	0.66 ± 0.031d	0.41 ± 0.024ab	0.04 ± 0.002d

ND, Not Detected; CAP, Capsaicin; DHC, Dihydrocapsaicin; n-DHC, Nordihydrocapsaicin. Different letters in each column of the sample analysis indicate significant differences between results at the 95% confidence level according to Tukey’s test. *Indicates the significant differences between results at the 95% confidence level according to Dunn’s test.

The concentrations of CAP, DHC, and n-DHC ranged from 0.01 to 3.75 mg/g, 0.01 to 2.70 mg/g, and 0.00 to 0.43 mg/g, respectively. The capsaicinoids concentration scale runs from Cristal > Hungarian Wax > Cacho de Cabra > ripe Cristal > Bell pepper.

The pungency index of peppers has been expressed in Scoville Heat Units (SHUs) as previously detailed elsewhere (Costa et al., 2022b). SHU represents the number of dilutions in water required for a sample to lose its pungency sensation. The pungency classification levels on the SHU are: non-pungent (0–700 SHU), mildly pungent (700–3000 SHU), moderately pungent (3000–25,000 SHU), highly pungent (25,000–80,000 SHU), and very highly pungent (>80,000 SHU).

As expected, in the present study, varieties containing higher amounts of CAP and DHC resulted in higher SHU values, since capsaicinoids’ concentrations are associated with the degree of pungency of chilli pepper (Saini et al., 2021). As previously reported by other authors, CAP and DHC are the two major capsaicinoids, responsible for up to 90% of the total pungency of pepper fruits (Fayos et al., 2017; Olguín-Rojas et al., 2019; Vázquez-Espinosa et al., 2023).

The Cristal and Hungarian Wax variety has the highest amount of CAP, DHC, and n-DHC (**Table 1**). The Cristal pepper is one of the medium-hot chilli varieties with a Scoville value of 30,000 to 50,000 SHU; whilst Hungarian Hot Wax’ belongs to the medium to low burning chillies, with a Scoville value of 5,000 to 10,000 SHU (Costa et al., 2019a).

The Cacho de Cabra is a Chilean variety of *C. annuum* that is generally recognised as a medium-hot pepper (FIA, 2010). To the best of our knowledge, this study represents the first record of the quantification of capsaicinoids in this chilli landrace.

The Cristal pepper variety presents considerable differences in terms of capsaicinoid content depending on the ripeness stage. The ripe Cristal pepper showed concentrations of CAP, DHC, and n-DHC lower than all the pungent chilli pepper varieties and slightly higher than the bell pepper.

Previous studies have analysed the concentration of capsaicinoids in ripe and unripe chilli peppers (Menichini et al., 2009; Vázquez-Espinosa et al., 2023), and in the different parts of the chilli pepper fruit throughout the ripening process (Contreras-Padilla and Yahia, 1998; Estrada and Bernal, 2000; Barbero et al., 2014; Fayos et al., 2017; Olguín-Rojas et al., 2019; Vázquez-Espinosa et al., 2023).

Overall, capsaicinoids begin to accumulate in the early stages of fruit development, increasing during ripening until they reach a maximum concentration, usually between days 40 and 60 post-anthesis (dpa). Due to the peroxidase’s enzyme action, at this stage, there is a rapid turnaround, with capsaicinoids degradation (c.a. 30 and 90%) (Estrada and Bernal, 2000; Barbero et al., 2014; Fayos et al., 2017; Olguín-Rojas et al., 2019; Vázquez-Espinosa et al., 2023).

Contrasting results have already previously been reported, with capsaicinoids remaining constant and increasing after 60 dpa, and showing oscillations in capsaicinoids’ content for the fruit of the same age, harvested at the same time and in the same position on the plant (Mueller-Seitz et al., 2008; Fayos et al., 2017; Vázquez-Espinosa et al., 2023). These differences in capsaicinoids’ content can be explained by variations in genotype, environmental conditions (e.g., drought), and part of the fruit analysed (e.g., pericarp, placenta, and seed) (Vera-Guzmán et al., 2017; Vázquez-Espinosa et al., 2023).

The lowest amount of CAP and DHC were detected in Bell pepper, a low-pungency pepper. A mutation in the key genes (e.g., *Pun1*, *pAMT*) involved in capsaicinoids’ biosynthesis leads to low or no pungency in chilli peppers (Tsurumaki and Sasanuma, 2019). Overall, capsinoids (e.g., capsiate, dihydrocapsiate, and nordihydrocapsiate), which is an analogous group to capsaicinoids, are prevalent in mild pepper (Macho et al., 2003). Capsinoids have valuable pharmaceutical properties (e.g., antitumoral, antioxidant, and anti-obesity) with the advantage of being less toxic than capsaicinoids (Costa et al., 2022b).

### 3.2 Total phenolic compounds of *Capsicum* spp. extracts

Phenolic compounds (e.g., phenolic acids, flavonoids, lignans, and so forth) are secondary metabolites that are widely found in *Capsicum* plants (Liu et al., 2020; Anaya-Esparza et al., 2021). It has been shown that these compounds can promote human health, mainly due to their antioxidant activity, which is associated with a reduced risk of cancers, and cardiovascular and neurodegenerative disorders (Sova and Saso, 2020; Anaya-Esparza et al., 2021).

**Figure 2:**
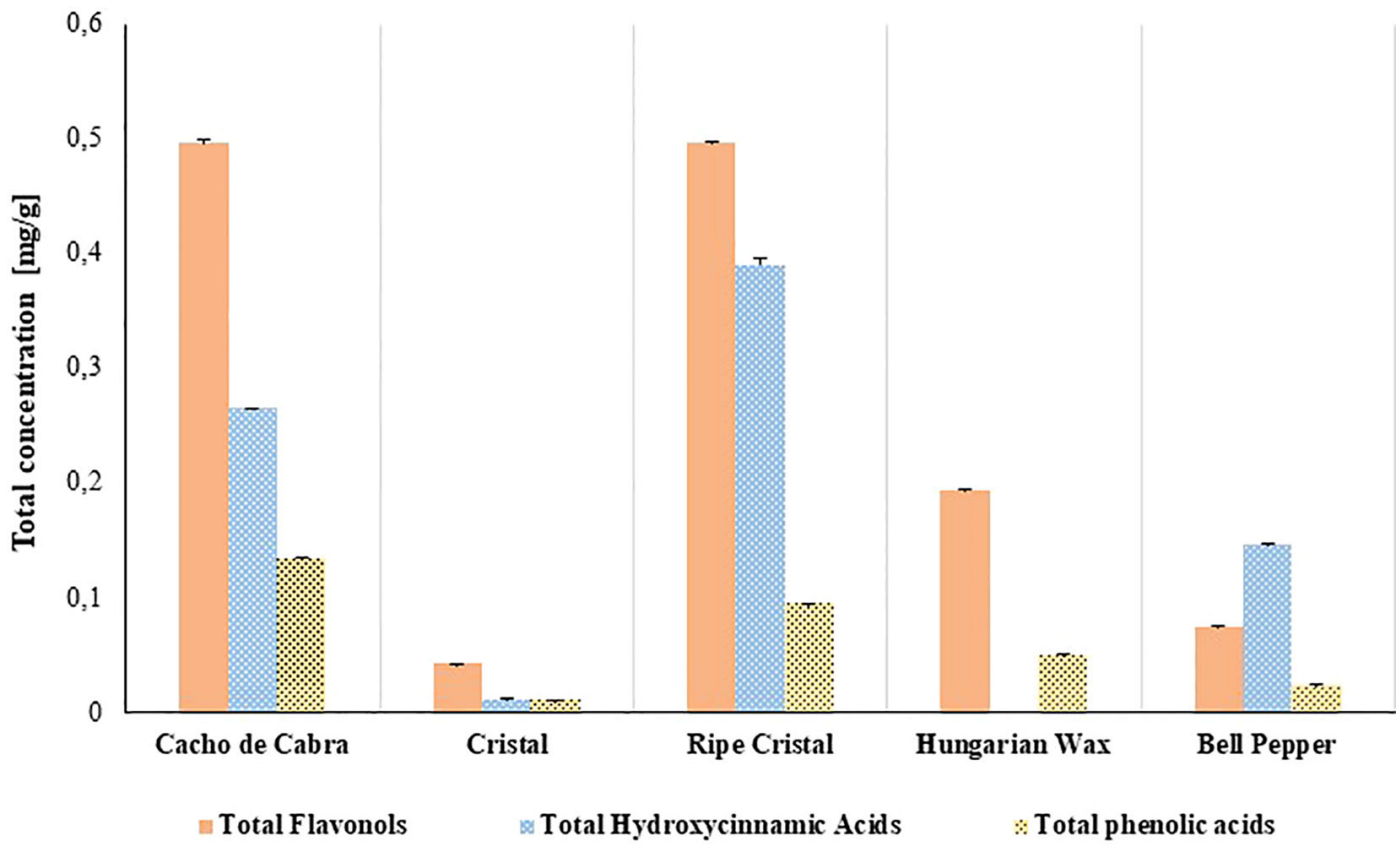
Phenolic compounds of *Capsicum* varieties determined by HPCL-DAD. Orange bars: Total flavanols content at 360 nm. Blue bars: Total content of hydroxycinnamic derivatives at 320 nm. Yellow bars: Total content of phenolic acids at 280 nm.

In the present study, the total hydroxycinnamic acids (THCA), total flavonols (TF) and total phenolic acids (TPA) of *Capsicum annuum* and *C. baccatum* varieties were assessed. There were noticeable differences in THCA, TF, and TPA values among the varieties analysed (**Figure 2**). The Cacho de Cabra and the ripe Cristal peppers showed the highest THCA, TF and TPA; while the lowest values were observed for the fresh Cristal pepper. The Hungarian Wax did not present derivatives of hydroxycinnamic acids.

Up to now, there has been little data on the chemical characterisation of the landraces and species of *Capsicum* cultivated in Chile (Lutz et al., 2015; Riquelme and Matiacevich, 2017; Muñoz-Concha et al., 2020). Muñoz-Concha et al. (2020) analysed the TPC, antioxidant capacity and total carotenoid content of chilli pepper Cacho de Cabra. The author reported a higher TPC value with 325 mg GAE/100 g. Similarly, Lutz et al. (2015), evaluated the total phenolic content and antioxidant capacity of vegetables grown in Chile. The sweet green pepper (*C. annuum*) and red pepper (*C. annuum* cv. Almuden) showed a TPC of 6.9 ± 0.04 mg GAE/g dry mass and 7.1 ± 0.1 mg GAE/g dry mass in fresh samples, respectively. In dehydrated samples, the TPC was 58.3 ± 0.8 mg GAE/g dry mass in green pepper and 62.6 ± 1.7 mg GAE/g dry mass in red pepper. The chemical characterisation of these cultivars is essential since a higher quantity of phytochemicals with nutritional value and a beneficial effect on both human and plant health could increase their commercial value.

Flavonols are a class of flavonoids, commonly isolated from *Capsicum* pepper. The results presented herein showed a high variation in flavonols’ values among the assessed samples, with the highest TF content in the Cacho de Cabra pepper and the ripe Cristal pepper (**Figure 2**).

The flavonol glycosides (e.g., quercetin-O-glycosides) are mainly found in *Capsicum* fruits. Marín et al. (2004) analysed different flavonoids in the pericarp of sweet peppers (*C. annuum* L. cv. Vergasa). According to the author, 23 flavonoids were detected and quantified by HPLC. The most abundant compounds were both flavonols the quercetin-3-O-rhamnoside and the luteolin 7-O- (2-apiosyl-6-malonyl) glucoside, which represented 41% of the total flavonoids.

Previous studies suggested that the flavonoid composition of the pepper fruit decreased during ripening, with the same trend for total phenols and total hydroxycinnamic (Sukrasno and Yeoman, 1993; Howard et al., 2000; Navarro et al., 2006; Ghasemnezhad et al., 2011; Martí et al., 2011); while others reported the opposite situation (Lee et al., 1995; Fox et al., 2005; Serrano et al., 2010; Shaha et al., 2013; Ribes-Moya et al., 2020). Most of these studies analysed a limited number of chilli pepper varieties, which can contribute to contradictory results.

Ribes-Moya et al. (2020) accessed a set of 14 *Capsicum* specimens, including *C. annuum* (12), *C. chinense* (1) and *C. baccatum* (1), and analysed the effect of organic conditions, ripeness stage, genotype and its interactions on the accumulation of antioxidant phenolic compounds. According to the authors, the ripening process increases the level of flavonoids, pointing out that the fluctuation in flavonoid content during ripening can vary depending on the type of flavonoid. These findings are in agreement with a recent study, which reported that the ripening process favoured the accumulation of total phenolics in a collection of 37 *Capsicum* accessions (Ribes-Moya et al., 2018).

The effect of chilli pepper varieties (Butcher et al., 2012; Fratianni et al., 2020), environments (Butcher et al., 2012; Meckelmann et al., 2015), ripening stages (Navarro et al., 2006; Martí et al., 2011; Shaha et al., 2013; Hamed et al., 2019), agricultural cultivation practices (e.g., organic/conventional) (Ribes-Moya et al., 2018, 2020), and harvests and post-harvest processing (e.g., drying process, temperature) can affect TPC and TFC concentrations in chilli peppers (Tvrzník et al., 2009; Ghasemnezhad et al., 2011; Shotorbani et al., 2013; Téllez-Pérez et al., 2013; Hamed et al., 2019).

### 3.3 Total phenols and antioxidant capacity

The content of total phenols and antioxidant activity in the *Capsicum* varieties were evaluated with spectrophotometric methods (**Table 2**). The total content of phenols ranges from 1.30 to 6.36 mg/g. These values are within the range reported for varieties of *Capsicum annuum* L, between 0.5 mg/g and 49 mg/g (Kavuncuoglu et al., 2021; Franczuk et al., 2023).

**Table 2:** Total phenolic compound concentrations and antioxidant activity of *Capsicum* spp. varieties.

Variety	Total phenols (GAE mg/g)	DPPH (TE µmol/g)*	TEAC (TE µmol/g)*	CUPRAC (TE µmol/g)*
Cacho de Cabra	4.69 ± 0.070b	32.23 ± 1.38b	27.44 ± 0.11b	40.85 ± 1.81b
Cristal	1.30 ± 0.019e	1.26 ± 0.16e	2.89 ± 0.14e	3.28 ± 0.18d
ripe Cristal	4.08 ± 0.022c	8.00 ± 0.73c	12.13 ± 0.18c	16.88 ± 2.08c
Hungarian Wax	3.69 ± 0.055d	6.04 ± 0.69d	7.81 ± 0.34d	13.22 ± 2.39c
Bell Pepper	6.36 ± 0.234a	54.74 ± 5.20a	45.20 ± 0.76a	58.25 ± 4.01a

GAE, Gallic acid equivalents; TE, Trolox equivalents. Different letters in each column indicate significant differences between results at the 95% confidence level according to Tukey’s test. *Indicates that the significant differences were obtained by logarithmic transformation of the original values shown in the table.

In the present study, the Bell Pepper extract showed the higher antioxidant activity, followed by Cacho de Cabra, ripe Cristal, Hungarian Wax and Cristal pepper extract (**Table 2**). This trend was also obtained for total phenol content (TPC), suggesting a correlation between the TPC and the antioxidant activity of the *Capsicum* peppers analysed.

These findings are in agreement with previous studies which indicated that *Capsicum* extracts with a high TPC had a stronger free radical scavenging effect (Loizzo et al., 2015; Sandoval-Castro et al., 2017; Liu et al., 2020).

*Capsicum* pepper is widely known as a food source of antioxidant compounds (e.g., ascorbic acid, capsaicinoids, carotenoids) (Gupta et al., 2021; Leng et al., 2022). Among these, phenolic compounds (e.g., hydroxycianmates, flavonols and flavones) play a major role in scavenging free radicals (Leng et al., 2022). The antioxidant action of phenolic compounds, which can prevent oxidative damage caused by reactive oxygen species (ROS), can employ different mechanisms, such as chelation of metal ions, elimination of free radicals and disintegration of peroxides (Leng et al., 2022).

For the agricultural sector, prospecting *Capsicum* molecules and their possible application as exogenous antioxidants is a relevant field that has been little explored. The application of extracts rich in capsaicinoids and phenolic compounds with antioxidant activity could be applied from seeds to cultivars, conferring not only antifungal protection but also improvements in their physiological parameters (e.g., photosynthetic capacity, stomatal conductance, transpiration rates, resistance to stressful conditions). Some studies have analysed the addition of exogenous antioxidant molecules (Shalata and Neumann, 2001; Shi et al., 2015; Singh et al., 2015; Queiroz et al., 2023). However, the possible effects of exogenous treatment with capsaicinoids and *Capsicum* molecules have not yet been fully studied. *In vivo* tests must be carried out to ensure improvement activity and avoid adverse effects (Barchenger and Bosland, 2016).

As shown in the present study, phenolic content and antioxidant activity can vary according to the *Capsicum* species and landrace analysed (Butcher et al., 2012; Fratianni et al., 2020). Similar results have been reported in the literature, highlighting the importance of understanding the composition of molecules with antioxidant activity in *Capsicum* spp.

In addition to genetic factors, the conditions of the growing season and the post-harvest stage (e.g., handling, packaging and processing) can significantly contribute to different levels of antioxidant compounds among species and ecotypes (Tvrzník et al., 2009; Ghasemnezhad et al., 2011; Shotorbani et al., 2013; Téllez-Pérez et al., 2013; Ribes-Moya et al., 2018; Hamed et al., 2019; Ribes-Moya et al., 2020).

### 3.4 MFCs and well diffusion method tests

The aqueous extracts of *Capsicum* landraces of Cacho de Cabra, Bell pepper, Hungarian Wax, Cristal, and ripe Cristal fruits were tested at 0.39, 0.78, 1.56, 3.1, 6.25, 12.5, 25, 50, 100 and 200 mg/mL against the crop pathogens and/or the post-harvest spoilage fungi *Alternaria* sp., *Fusarium oxysporum*, *F*. *verticillioides*, *Aspergillus niger*, *A. dimorphicus*, *Penicillium citrinum* and *Rhizopus arrhizus*. In addition, for the standards, final concentrations of 0.48, 0.97, 1.9, 3.9, 7.81, 15.62, 31.25, 62.5, 125 and 250 µg/mL were assessed. Overall, based on the results of MFC tests, all of these fungal strains presented differences in their micro-morphological characteristics when treated with aqueous extracts of *Capsicum* spp. at concentration between 50 and 100 mg/mL; while for standards CAP and DHC, the fungal strains presented differences in their micro-morphological characteristics when treated with concentrations of among 62.5 µg/mL 125 and 250 µg/mL.

**Figure 3:**
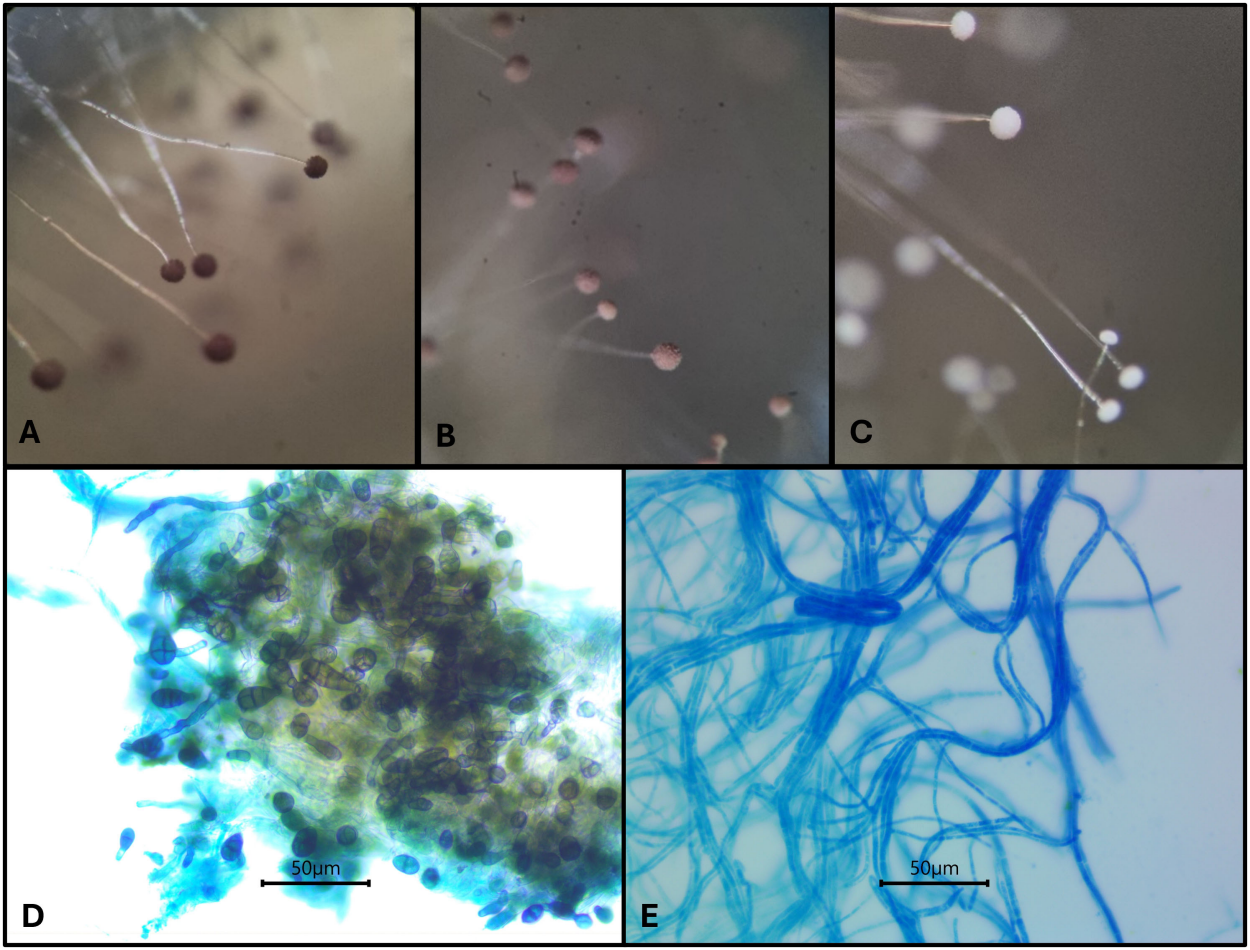
Impact of CAP and pepper pod extracts on the micro-morphological characteristics of *Aspergillus niger* MUM05.11 **(A to C)** and *Alternaria* sp. UFRO17.178 **(D, E)**. For *A niger* MUM05.11: **(A)** control; **(B)** and **(C)** treatments with CAP at 62.5 μg/mL and 250 μg/mL, respectively. For *Alternaria* sp. UFRO17.178: **(D)** control and **(E)** treatments with aqueous extract of *C. annuum* Cacho de Cabra variety (150 mg/mL).

According to the results obtained herein, modifications of the macro and micromorphological characteristics in filamentous fungi strains were observed. After treatment with *Capsicum* aqueous extracts, the fungal strains *Aspergillus niger* MUM05.11, *A. dimorphicus* UFRO17.168, *Fusarium oxysporum* ATCC48112, *F. verticillioides* MUM16.143, *Penicillium citrinum* UFRO17.97 and *Rhizopus arrhizus* MUM16.05 produced mycelia with a thin, fragile and easily breakable structure (**Figures 3A-E**).

The conidiophores of the strain *A. niger* MUM05.11 became fragile presenting easily breakable structures and loss of pigmentation (**Figures 3A-C**). These changes are reflected from 30 – 40 µg/mL concentration (data not shown), being more pronounced at 62.5 µg/mL for the CAP standard (**Figure 3C**). For the pepper extracts, these fungistatic effects were observed at 50 mg/mL, where the most drastic change was observed over 100 mg/mL (**Supplementary Figure 1**).

Previous studies have shown CAP from different *Capsicum* species, even at lower concentrations, inhibited the growth of fungi (Soumya and Nair, 2012; Buitimea-Cantúa et al., 2018). Whilst others point out that the fungi in the presence of CAP, regardless to hyphae growth inhibition, showed consistent morpho-physiological changes (Behbehani et al., 2023).

The concentration used for the susceptibility tests in a solid medium was determined based on the results and the concentration of capsaicinoids present in each *Capsicum* aqueous extract. The standards of capsaicinoids were set at 100 µg/mL and the *Capsicum* aqueous extracts at 150 mg/mL.

For *Alternaria* sp. UFRO17.178 treated with aqueous extract of *C. annuum* Cacho de Cabra variety (150 mg/mL) a micro-morphological change was observed, pointing out to the occurrence of conidiogenesis inhibition. This fungal strain was not able to produce conidiophores, as it can be observed as shown in **Figure 3E**.

According to the data obtained herein, controlling fungal growth and reproduction is an important achievement of using these pepper pod extracts, once they do not kill fungal biodiversity, but could control their growth, reproduction as a pathogen and/or spoilage fungi in the field.

For the *Alternaria* genus, the conidiogenesis inhibition has already previously been reported for other antifungal molecules (Romero-Cortes et al., 2019; Zhang et al., 2020; Li et al., 2021). Soylu and Kose (2015) evaluated the effect of essential oils of *Origanum onites* (oregano), *Thymbra spicata* (thyme) and *Foeniculum vulgare* (fennel) against *Alternaria alternata*. According to the authors, a total conidiogenesis inhibition of *A. alternata* was observed under the applied treatments. In addition, the effect of CAP on conidial germination of *Colletotrichum capsici* has also been reported (Kraikruan et al., 2008). In this case, *C. capsici* conidiogenesis was completely inhibited when the fungus was treated with capsaicin at a concentration of 100 and 200 mg/L on PDA medium.

In addition, in the present study the antifungal activity was assessed based on the fungal growth on SDA medium supplemented with amphotericin B, CAP or DHC. According to the results obtained herein, fungal inhibition ranged from 43 to 64% for amphotericin B; 20 to 33% for CAP; and, 7 to 18% for DHC (**Table 3**). In contrast, in the positive control (SDA medium + fungi) fungi strains grown completely on Petri dish.

**Table 3:** Antifungal activity of *Capsicum* extracts against different fungal species.

Fungal species	Inhibition ratio (%)							
	AMB	CAP	DHC	Cacho de Cabra	Cristal	Ripe Cristal	Hungarian Wax	Bell Pepper
*A.* ***dimorphicus* UFRO17.168**	47	20	13	−	−	−	−	−
*A.* ***niger* MUM05.11**	62	25	18	15	18	−	4	−
*F.* ***oxysporum* ATCC48112**	47	26	16	5	5	−	7	−
*F.* ***verticillioides* MUM16.143**	46	20	15	−	−	−	−	−
*P.* ***citrinum* UFRO17.97**	43	25	7	−	−	−	−	−
*R.* ***arrhizus* MUM16.05**	50	25	13	5	13	−	−	−
*Alternaria* **sp. UFRO17.178**	64	33	15	3	9	−	−	−

−: No inhibition ratio observed at macroscopic level.

Regarding chilli pepper extracts, the Hungarian Wax extracts produced a slight growth inhibition of *A. niger* MUM05.11 and *F. oxysporum* ATCC48112 (**Table 3**); while the Cacho de Cabra and Cristal extracts inhibited the *A. niger* MUM05.11, *F. oxysporum* ATCC48112, *R. arrhizus* MUM16.05, and *Alternaria* sp. UFRO17.178 growths at a lower level than that obtained by AMB, CAP, and DHC. The ripe Cristal and fresh Bell peppers showed no antifungal activity against any of the analysed fungal strains.

*Capsicum* spp. extracts did not inhibit both growth and conidiogenesis of the fungal strains *A. dimorphicus* UFRO17.168, *F. verticillioides* MUM16.143 and *P. citrinum* UFRO17.97. However, it was observed that these strains shown fragile and easily breakable mycelia after treatments.

Overall, the results presented herein show noticeable microscopic changes, in the presence or absence of mycelial growth inhibition, indicating a fungistatic effect of the extracts analysed. This was observed through the presence of fungal fragile structures, loss of pigmentation, a variation in the size of the reproductive structures and the number of conidia.

Similarly, Singh and Chittenden (2008) observed drastic morphological changes in the fungal strains *Leptographium procerum* and *Sphaeropsis sapinea* that had been treated with chilli pepper extracts, obtaining more compact mycelia and hyperbranching of the hyphae.

The fungistatic activities of both *Capsicum* spp. extracts and purified capsaicinoids have been demonstrated *in vitro*. The growth rate of both phytopathogenic fungi (e.g., *Botrytis cinerea*, *Cladosporium cucumerinum*, *Colletotrichum gloeosporioides*, *Fusarium oxysporum*, *Fusarium* sp., *Penicillium digitatum*, *P. expansum, Rhizoctonia solani*) and food spoilage fungi, such as mycotoxigenic strains (e.g., *Aspergillus flavus*, *A. niger*, *Aspergillus parasiticus*) has been modified in the presence of capsaicinoid compounds (Singh and Chittenden, 2008; Singh et al., 2011; Moreno-Limón et al., 2012; Soumya and Nair, 2012; Ziglio and Gonçalves, 2014; Fieira et al., 2013; Kollia et al., 2019; Buitimea-Cantúa et al., 2020; Saini et al., 2021; Vázquez-Fuentes et al., 2021; Nidiry, 2022).

Mostly of studies with *Capsicum* extracts showed a fungistatic effect, i.e., *Capsicum* extracts were unable to kill the fungus, but does prevent continuing fungi growth and/or reproduction (Singh and Chittenden, 2008; Moreno-Limón et al., 2012; Soumya and Nair, 2012; Ziglio and Gonçalves, 2014; Fieira et al., 2013; Kollia et al., 2019; Buitimea-Cantúa et al., 2020; Saini et al., 2021; Vázquez-Fuentes et al., 2021; Nidiry, 2022). The fungistatic potential of *Capsicum* extracts is an important characteristic, since they will not kill fungal biodiversity, but can control its growth and reproduction in the field.

### 3.5 Toxicity tests

*Galleria mellonella* larvae are widely used as a model to assess the *in vivo* toxicity of antifungal compounds, including pure substances and plant extracts (Curtis et al., 2022). The data presented herein show that the group of larvae inoculated with extracts of Bell pepper (2000mg/kg) and Cristal (2000mg//kg) had an 80% survival rate at the end of the experiment; while the extracts of Cacho de Cabra (2000 mg/kg) and ripe Cristal (20 mg/kg) had a 90% survival rate at the end of the experiment.

The group of larvae inoculated with extracts of Hungarian Wax (2000 mg/kg), showed a 100% survival rate. This indicates that the extracts of *Capsicum* spp. analysed in the present study were not toxic for the larvae. *Galleria mellonella* larvae untreated and water-injected survived until the end of the study. The group inoculated with 99.9% ethanol began to die on day 2 (**Supplementary Figure 2**).

## 4 Conclusions

The fresh Cristal and Hungarian Wax variety presented the highest amount of CAP, DHC, and n-DHC, followed by Cacho de Cabra, ripe Cristal and Bell pepper. The higher amounts of CAP and DHC are related to higher SHU values since capsaicinoids are associated with the degree of chilli pepper pungency.

Bell Pepper had the highest phenol content and highest values in all antioxidant activity measurement methods. The antioxidant phytochemicals of *Capsicum* have exhibited potential health benefits for the human body. An in-depth phytochemical profile of *Capsicum* species produced, consumed and marketed in Chile, especially local landraces, should be carried out. From this information, agronomic parameters (e.g., soil, irrigation, collection period, storage time) that affect the concentration of these compounds can be optimised to increase the pharmaceutical and nutritional value of *Capsicum* pepper, adding value to this commodity.

The extracts of *Capsicum* spp. show a slight fungal growth inhibition against *A. niger* MUM05.11, *F. oxysporum* ATCC48112, *R. arrhizus* MUM16.05 and *Alternaria* sp. UFRO17.178. Despite the absence of mycelial growth inhibition, 4 out of 5 *Capsicum* extracts assessed presented fungistatic effect. Noticeable changes in the fungal strains, such as the presence of fragile fungal structures, loss of pigmentation, variation in reproductive structure size and the number of conidia were observed.

Fresh extracts of Cacho de Cabra, Cristal and Hungarian Wax varieties presented fungistatic effect against all of the fungal strains assessed. In addition, ripened Cristal presented fungistatic effect against *Aspergillus niger* MUM05.11, *A. dimorphicus* UFRO17.168 and *Penicillium citrinum* UFRO17.97. Future field work should be carried out to validate the *in vitro* results.

The non-toxicity of *Capsicum* extracts together with their fungistatic capability underline their potential as an environmentally friendly biotechnology, especially in the agri-food sector, to control the growth and reproduction of fungi without posing a risk to non-target biodiversity. Added to its fungistatic activity, the possible effects of exogenous treatment with *Capsicum* antioxidant molecules on the cultivars’ development should be researched in depth.

## Data Availability

The original contributions presented in the study are included in the article/**Supplementary Material**. Further inquiries can be directed to the corresponding author.
