# Supplementary material for: Chemical composition and antifungal activity of Capsicum pepper aqueous extracts against plant pathogens and food spoilage fungi
Source: Front Cell Infect Microbiol. 2024 Oct 3;14:1451287. doi: 10.3389/fcimb.2024.1451287 (PMC11484085; doi:10.3389/fcimb.2024.1451287)
Supplement: Supplementary file 1 [file DataSheet1.pdf]

Supplementary Material

1. Supplementary Figures

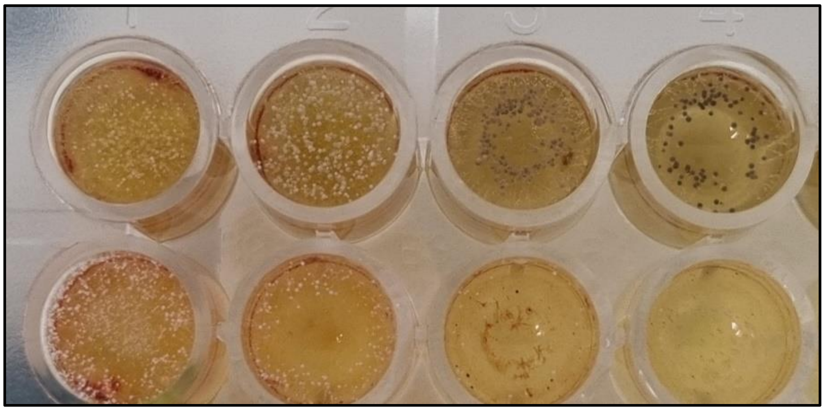

Supplementary Figure 1. *Aspergillus niger* MUM05.11 (upper part) from left to right; treatment with CAP at 250 ug/mL, 125 ug/mL and 60 ug/mL respectively and control (only RPMI 1640 medium).

A

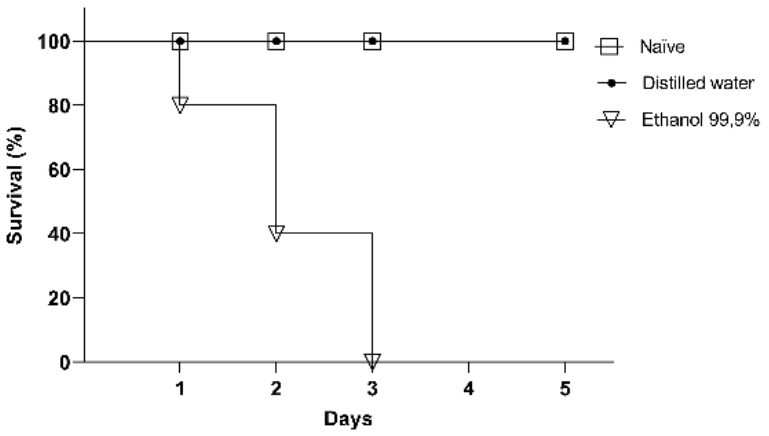
Survival (%)
100
80
60
40
20
0
1
2
3
4
5
Days
Naïve
Distilled water
Ethanol 99,9%

B

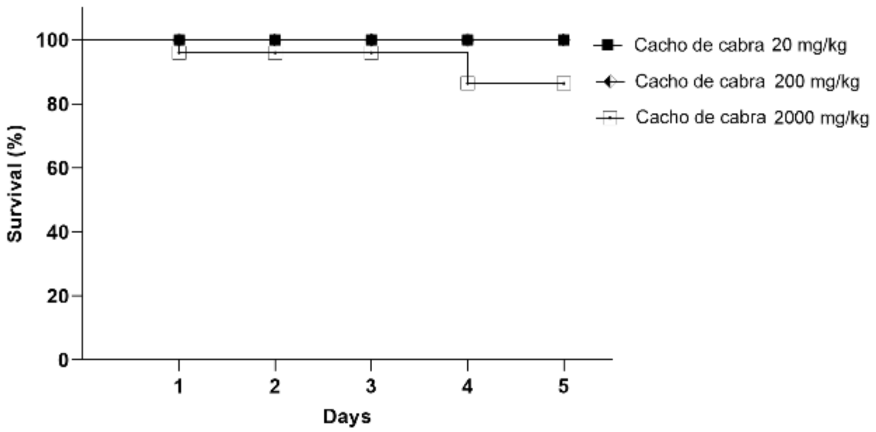
Survival (%)
100
80
60
40
20
0
1
2
3
4
5
Days
Cacho de cabra 20 mg/kg
Cacho de cabra 200 mg/kg
Cacho de cabra 2000 mg/kg

C

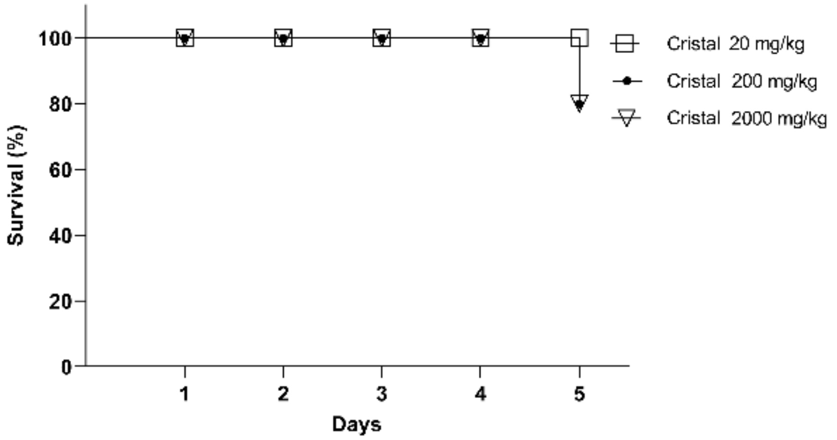
Survival (%)
100
80
60
40
20
0
1
2
3
4
5
Days
Cristal 20 mg/kg
Cristal 200 mg/kg
Cristal 2000 mg/kg

D

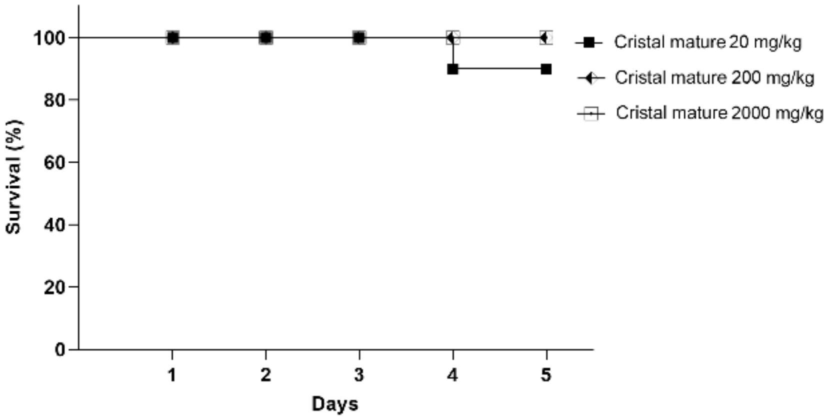

E

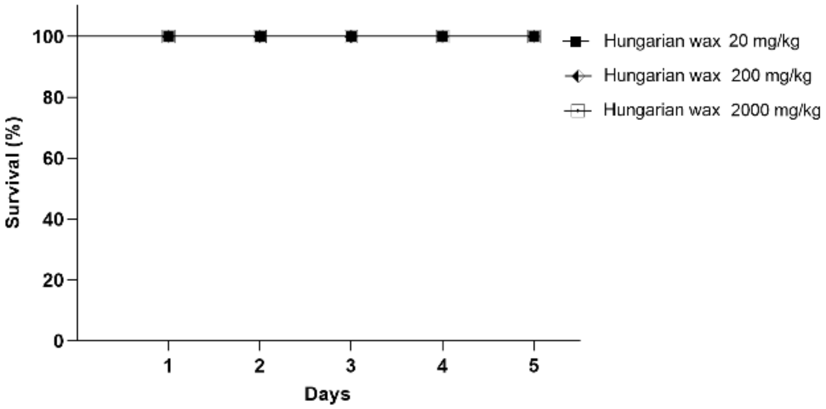

F

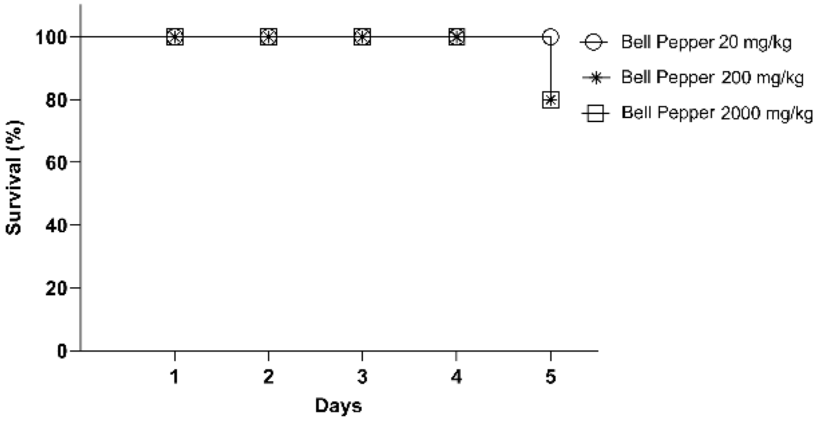

Supplementary Figure 2. Kaplan-Meier survival curve of *Galleria mellonella* larvae injected with aqueous extracts of *Capsicum annuum* and *C. baccatum* varieties. (A) control; (B) treatments with aqueous extract of *C. annuum* Cacho de Cabra; (C) treatments with aqueous extract of *C. baccatum* Cristal; (D) treatments with aqueous extract of mature Cristal; (E) treatments with aqueous extract of *C. annuum* Hungarian Wax; (F) treatments with aqueous extract of *C. annuum* Bell pepper.
